# Molecular mechanisms of enzalutamide resistance in prostate cancer

**DOI:** 10.20517/cdr.2019.25

**Published:** 2019-06-19

**Authors:** Eliot B. Blatt, Ganesh V. Raj

**Affiliations:** ^1^Department of Urology, University of Texas Southwestern Medical Center, Dallas, TX 75390, USA.; ^2^Department of Pharmacology, University of Texas Southwestern Medical Center, Dallas, TX 75390, USA.

**Keywords:** Drug resistant cancers, resistance modulation, biomarkers of drug responsiveness, targeted therapy resistance

## Abstract

An estimated 30,000 men in the United States will die of metastatic prostate cancer (PCa) each year due to the development of therapy resistance, most notably resistance to second-generation antiandrogen enzalutamide. The vast majority of PCa is driven by the androgen receptor (AR). Enzalutamide is an AR antagonist, which extends patient survival and is widely used in the clinic for the treatment of castration-resistant prostate cancer (CRPC); however, many patients will have primary or develop acquired resistance and continue to progress. Characterization of the molecular mechanisms of enzalutamide resistance provides insight into potentially efficacious therapies for enzalutamide-resistant CRPC (ER-CRPC). Understanding these mechanisms is critical for the identification of biomarkers predictive of therapy resistance and the development of therapeutic strategies to target ER-CRPC.

## Introduction

Nearly 30,000 men in the United States will die of metastatic prostate cancer (PCa) in 2019^[1,2]^. Despite the introduction of seven new Food and Drug Administration-approved therapeutic agents since 2007, metastatic PCa is incurable. The mainstay of treatment for metastatic PCa remains androgen deprivation therapy (ADT), via pharmacological or surgical castration. The majority of patients will initially respond to ADT; however, a significant proportion becomes therapy-resistant and develops castration-resistant prostate cancer (CRPC)^[3,4]^.

**Figure 1:**
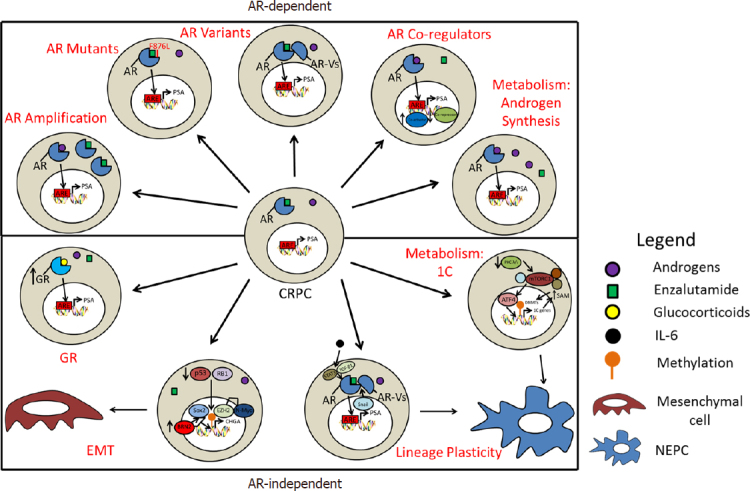
Model of androgen receptor (AR)-dependent and AR-independent mechanisms that enable a castration-resistant prostate cancer (CRPC) to become an enzalutamide resistant-CRPC. EMT: epithelial-mesenchymal transition; GR: glucocorticoid receptor; 1C: one-carbon; NEPC: neuroendocrine prostate cancer

The androgen receptor (AR) is a steroid hormone nuclear receptor that drives the vast majority of PCa. CRPC remains dependent on AR signaling and second-generation antiandrogens, such as the AR antagonist enzalutamide or the androgen synthesis inhibitor abiraterone, extend the overall survival of metastatic CRPC patients by a median of 4-5 months^[5,6]^. However, primary or acquired resistance to these agents is common with cancer recurrence and progression. Multiple mechanisms of enzalutamide resistance have been defined and offer insights into further therapeutic strategies against enzalutamide-resistant CRPC (ER-CRPC)^[7-9]^. In this review, we present a critical evaluation of the defined molecular mechanisms, potential biomarkers, and therapeutic options for ER-CRPC.

## AR amplification

For receptor-ligand interactions, the relative concentrations of receptors, agonists, and antagonists in a cell can dictate whether antagonists will be efficacious in inhibiting their target. Stoichiometric ratios of AR, enzalutamide, and androgens determine whether AR is bound to agonist or antagonist, and whether AR signaling is active. Sequencing of CRPC and ER-CRPC patient tumors demonstrates that 70% of patients have significant AR pathway alterations, with the vast majority containing AR gene amplifications^[10,11]^. Thus, AR antagonists, like enzalutamide, apalutamide, or darolutamide, may be effectively unable to antagonize all AR proteins in this context. Furthermore, since CRPC and ER-CRPC tumors may synthesize their own androgens, sustained or enhanced AR signaling is noted, even under castrate-level serum androgen conditions^[12]^. Thus, AR amplification and increased AR signaling are robust mechanisms of resistance to first- and second-generation antiandrogens^[10,11,13-16]^.

## AR mutants

AR point mutations that convert first-generation antiandrogens, such as flutamide and bicalutamide, from antagonists to agonists have been well-characterized. These mutations are in the ligand binding domain (LBD), and include T877A and W741C point mutations, which mediate resistance to flutamide and bicalutamide, respectively^[13-16]^. Similarly, resistance to enzalutamide has been modeled to be driven by an AR F876L point mutation. This mutation has been infrequently identified in ER-CRPC samples and confers agonistic properties to both enzalutamide and apalutamide^[17-19]^. Importantly, since these mutations are specific for each antiandrogen, other antiandrogens can have activity against AR. For example, CRPC cells with T877A and W741C mutations are resistant to flutamide and bicalutamide, respectively, but are sensitive to enzalutamide and apalutamide^[17]^. While AR mutations can confer resistance to enzalutamide, the low observed prevalence of this mutation in ER-CRPC patients does not support a significant clinical role for this mutation in enzalutamide resistance.

## AR variants

There are a large number of known AR splice variants (AR-Vs)^[20-24]^. AR variants primarily appear after the selection pressure of castration and more frequently after enzalutamide and abiraterone treatment^[20-22,24-31]^. The most common AR-V is AR-V7 (AR3), which contains exons 1, 2, 3, and a cryptic exon 3b but lacks the LBD^[21,26,27]^. AR-Vs can be constitutively active, and detection of AR-Vs in CRPC correlate with poor survival, progression, and therapy resistance^[20-22,24-31]^. Assays to detect AR-Vs in circulating tumor cells are commercially available and predict therapy response to enzalutamide and abiraterone^[25]^. AR-Vs can substitute for the full-length AR (AR-FL) and can bind to canonical androgen responsive elements (AREs) on the chromatin, heterodimerize with AR-FL, and drive transcription from AREs^[31-34]^. AR-Vs have been shown to be capable of independently driving transcription, cell proliferation, and DNA repair in CRPC^[35]^. However, AR-Vs are rarely seen without significant amplification of AR-FL, and its activity may be dependent on full-length AR^[31,32,36]^.

Importantly, the ability of AR-Vs to independently drive ER-CRPC has not been proven. *In vitro* studies with knockdown of variants in CRPC cell lines that express both AR-FL and AR-Vs indicate that AR-V expression confers a distinct growth advantage, when treated with antiandrogens^[31-34,37]^. Treatment with niclosamide inhibits AR-V7 recruitment to AR target genes, reduces AR-V7 protein levels in a proteasome-dependent manner, and re-sensitizes ER-CRPC cells to enzalutamide^[38]^. Induction of AR-Vs through NF-κB2/p52 can also enhance enzalutamide resistance^[39-41]^. While these data suggest that AR-Vs can contribute to ER-CRPC growth and can mediate resistance to enzalutamide, they do not definitively establish that AR-Vs drive resistance to enzalutamide.

The true contribution of AR-Vs in driving resistance can only be clearly established from drugs that specifically target AR-Vs and not AR-FL; however, no such agent has been developed. Since AR-FL and AR-Vs share a common N-terminal domain (NTD), compounds designed to target the NTD will target both AR-FL and AR-Vs. For example, ESSA Pharma’s EPI-506, which was designed to target the AR NTD, could target both AR-Vs and AR-FL, but demonstrated no activity against enzalutamide- and abiraterone-resistant CRPC patients in Phase I clinical trials^[42]^. Ongoing clinical trials with AR degraders will also likely target AR-FL and AR-Vs, and are unlikely to establish AR-Vs as independent molecular drivers of ER-CRPC.

## AR co-regulators

Upon binding androgens and activation, AR alters its protein interactome, translocates to the nucleus, and regulates a pro-proliferative transcriptional program. AR interacts with a number of co-activators to enhance transcription, like members of the p160 steroid receptor coactivator (SRC) family, histone acetyltransferase CBP/p300, and pioneer factor forkhead box A1 (FOXA1), and co-repressors to inhibit transcription, such as zinc finger and BTB domain containing 16 (ZBTB16)^[43-48]^. Modulating the expression of these cofactors can enhance or attenuate AR transcriptional activity. For example, alterations in expression of coactivators SRC-1, SRC-2, SRC-3, CBP/p300, and FOXA1 and co-repressor ZBTB16 are frequently seen CRPC and ER-CRPC patients and are associated with worse prognosis^[10,11,49-53]^. Since enzalutamide can modulate the recruitment of AR cofactors, altering the expression of co-activators or co-repressors in CPRC can potentially bypass the inhibitory effects of enzalutamide and confer resistance^[48,54,55]^.

## Glucocorticoid receptor

Like AR, the glucocorticoid receptor (GR) is a steroid hormone nuclear receptor, which binds DNA as a homodimer in an inverted repeat fashion^[56,57]^. Upon binding its ligand, GR transcriptionally activates a stress response program, which enhances the expression of anti-inflammatory genes and suppresses pro-inflammatory genes^[58-60]^. Overexpression of GR was noted in ER-CRPC and mediates enzalutamide resistance, where it may bypass the need for AR signaling^[61]^. Primary GR-dependent enzalutamide resistance may be observed in a subset of CRPC tumors that have increased basal expression of GR. Acquired GR-dependent enzalutamide resistance entails a de-repression mechanism, whereby AR normally inhibits GR expression, and enzalutamide enables enhanced GR expression by blocking AR signaling^[61]^. Interestingly, chromatin immunoprecipitation-sequencing (ChIP-seq) studies in enzalutamide-resistant cells identified GR binding over 50% of AR binding sites on the chromatin, with the strongest AR-regulated genes also being regulated by GR^[61]^. Thus, overexpressed GR functionally substitutes for AR. Therapeutic targeting of GR in ER-CRPC has been proposed, with at least two companies, ORIC Pharmaceuticals and Corcept Therapeutics, developing GR antagonists.

## Epithelial-mesenchymal transition

The epithelial-mesenchymal transition (EMT) is a process by which epithelial cells become more mesenchymal, a state characterized by increased invasive capacity, apoptotic resistance, and enhanced motility and metastatic potential^[62-65]^. Acute enzalutamide treatment induces EMT through a number of mechanisms, including increasing TGF-β1 expression and STAT3 activation, as well as Snail induction^[66-68]^. Metformin blocks enzalutamide-induced EMT and improves PCa sensitivity to enzalutamide^[66]^. In addition, autocrine IL-6 can facilitate CRPC growth and confers enzalutamide resistance mediated by STAT3 activation^[38]^. Furthermore, AR directly represses Snail transcription, and acute enzalutamide treatment enhances Snail expression and EMT^[67]^. Importantly, in models of chronic enzalutamide treatment, enzalutamide resistance can be mediated by Snail induction of both AR and AR-V7 expression, leading to increased AR signaling^[69,70]^. Whether in an AR-dependent or -independent manner, programs that enable cell transitions in response to selective pressures are important mechanisms of resistance to enzalutamide.

## Metabolic alterations

Most molecular mechanisms identified in ER-CRPC have focused on AR transcriptional regulation and maintenance of AR signaling, despite AR inhibition. Evaluations of the downstream programs that confer enzalutamide resistance often culminate in metabolic alterations, as the metabolic state governs whether cells will resist stress and proliferate.

### Intracrine androgen synthesis

Increased androgen synthesis can overwhelm the ability of enzalutamide to block AR signaling^[12]^. Intracrine androgen synthesis has recently been shown to confer enzalutamide resistance through upregulation of steroid synthesis genes, such as aldo-keto reductase family 1 member C3 (AKR1C3)^[12,30,71-75]^. AKR1C3 catalyzes the conversion of androstenedione and 5 α-androstanedione to testosterone and DHT, respectively, and is enriched in acquired and *de novo* ER-CRPC^[12,76,77]^. Increased AKR1C3 levels results in upregulated intracrine androgen synthesis and confers resistance to enzalutamide^[12]^.

### Hypoxia

An AR-independent mechanism of enzalutamide resistance involves hypoxia and the metabolic consequences of hypoxia-induced programs driven by hypoxia-inducible factor (HIF)^[78]^. PCa cells that are capable of stimulating hypoxia-induced survival programs through the upregulation of hypoxia response genes, such as glucose-6-phosphate isomerase (GPI), are clonally selected to become AR-independent and resistant to enzalutamide^[78]^. Under normoxic and normal androgenic conditions, AR enhances glycolysis and the pentose phosphate pathway (PPP)^[79,80]^. Under hypoxic and normal androgenic conditions, the PPP is slightly upregulated, AR inhibits GPI, and glycolysis is inhibited. However, under hypoxic and castrate conditions, the PPP is inhibited, GPI is upregulated, and glycolysis is stimulated^[78]^. PCa cells that are thus able to redirect glucose away from the PPP and toward glycolysis are able to evade stress and proliferate normally^[78]^. Glycolytic inhibitors, such as 2-deoxyglucose, may be useful in this context; however, toxicity is a concern due to lack of selectivity. Selective glycolytic inhibitors in development may be efficacious against some forms of ER-CRPC. Of note, GPI is preferentially overexpressed in neuroendocrine prostate cancer (NEPC) tumors, attesting to the importance of metabolic rewiring in driving neuroendocrine disease^[78]^.

### One-carbon metabolism

Increased reliance on serine and one-carbon (1C) metabolism promotes enzalutamide resistance in NEPC^[81]^. In CRPC cells, loss of protein kinase C (PKC) λ/ι allows cells to transition from a luminal, AR-dependent phenotype to a basal, AR-independent phenotype through enhanced one-carbon metabolism and resulting epigenetic changes^[81]^. This upregulation in one-carbon metabolism is dependent on mammalian target of rapamycin 1 (mTORC1) and cyclic AMP-dependent transcription factor 4 (ATF4) and culminates in an increase in S-adenosylmethionine (SAM), which supports epigenetic reprogramming (DNA methylation)^[81]^. Enhancer of zeste homolog 2 (EZH2) inhibition has been shown to reverse NEPC to a more AR-dependent state sensitive to antiandrogens, which demonstrates the potential efficacy of this strategy and further indicates the importance of epigenetic changes for the development of NEPC^[11,82]^. Additionally, DNA methylation inhibitors, such as decitabine, may be efficacious in targeting neuroendocrine disease. mTORC1 inhibitors, such as everolimus, may also show benefits in NEPC patients with a PKCλ/ι deficiency.

## Lineage plasticity

Lineage plasticity is a mechanism through which cells can acquire characteristics of a lineage that no longer requires a certain drug target^[83]^. With enzalutamide treatment, cells become AR-independent and therefore enzalutamide-resistant. In PCa, lineage plasticity is a state characterized by significant epigenetic changes, decreased AR signaling, and an increased expression of neuroendocrine and stem cell markers^[82-84]^.

### p53 and retinoblastoma 1 loss

Enzalutamide resistance can develop from loss of tumor suppressors tumor protein p53 (TP53) and retinoblastoma 1 (Rb1) and a downstream SRY-box 2 (SOX2)-driven shift^[83]^ .The proposed mechanism involves increasing cell plasticity, which confers resistance through lineage switching to an AR-independent, basal-like cell^[83]^. Similarly, loss of p53 and Rb1 creates a stem cell-like epigenetic environment due to derepression of EZH2 (and SOX2), which allows for adaptation to selective pressures, such as enzalutamide^[82]^.

### BRN2

Regulators of Sox2, like POU-domain transcription factor BRN2 (POU3F2), drive the emergence of NEPC and enzalutamide resistance^[84]^. BRN2 expression is inhibited by AR, is required for the expression of neuroendocrine markers, and expressed in NEPC. Enzalutamide derepresses AR inhibition of BRN2 in CRPC and enables BRN2-driven transdifferentiation into enzalutamide-resistant NEPC^[84]^. Furthermore, BRN2 regulates SOX2, and these proteins directly interact at the enhancers of neuronal genes and cooperate to drive a neuroendocrine phenotype^[84]^. Targeting BRN2 remains an attractive option for preventing lineage plasticity and the development of AR-independent PCa.

### N-Myc and EZH2

N-Myc overexpression is found in 5% of primary PCa patients, 20% of CRPC patients, and roughly 40% of NEPC patients^[85-87]^. N-Myc and EZH2 cooperate to drive transdifferentiation into NEPC and enzalutamide resistance^[86]^. N-Myc differentially regulates the DNA damage response in a context-dependent manner. Upregulation of N-Myc inhibits ataxia-telangiesctasia mutated (ATM), which allows PCa to become CRPC. In CRPC, overexpression of N-Myc with EZH2 blocks ATM inhibition, leading to ATM upregulation^[88]^ and the development of enzalutamide-resistant NEPC. Given the dependence of NEPC on epigenetic reprogramming and EZH2 in particular, targeting EZH2 may be an effective therapeutic option. While some EZH2 inhibitors have failed clinical trials [NCT01897571], other agents, such as Constellation Pharmaceuticals’ CPI-1205 and Daiichi-Sankyo’s DS-3201b, may offer hope for selected patients with NEPC [NCT03480646, NCT03110354].

## Conclusion

Discovering the molecular underpinnings of enzalutamide resistance has led to a greater understanding of the factors that drive progression and the heterogeneity that belies ER-CRPC. Ongoing studies will enable the identification of biomarkers predictive of therapy resistance and the development of targeted therapies to overcome therapeutic resistance.

## Declarations

### Copyright

© The Author(s) 2019.
